# Synthesis and Characterization of Highly Fluorinated Hydrophobic Rare–Earth Metal–Organic Frameworks (MOFs)

**DOI:** 10.3390/ma17174213

**Published:** 2024-08-26

**Authors:** Muhammad Abbas, Bhargavasairam Murari, Simin Sheybani, Monu Joy, Kenneth J. Balkus

**Affiliations:** Department of Chemistry and Biochemistry, The University of Texas at Dallas, 800 West Campbell Rd, Richardson, TX 75080, USA

**Keywords:** metal–organic frameworks, MOFs, hydrophobic materials, coordination polymers

## Abstract

Tuning a material’s hydrophobicity is desirable in several industrial applications, such as hydrocarbon storage, separation, selective CO_2_ capture, oil spill cleanup, and water purification. The introduction of fluorine into rare-earth (RE) metal–organic frameworks (MOFs) can make them hydrophobic. In this work, the linker bis(trifluoromethyl)terephthalic acid (TTA) was used to make highly fluorinated MOFs. The reaction of the TTA and RE^3+^ (RE: Y, Gd, or Eu) ions resulted in the primitive cubic structure (*pcu*) exhibiting RE dimer nodes (RE-TTA-*pcu*). The crystal structure of the RE-TTA-*pcu* was obtained. The use of the 2-fluorobenzoic acid in the synthesis resulted in fluorinated hexaclusters in the face-centered cubic (*fcu*) framework (RE-TTA-*fcu*), analogous to the UiO-66 MOF. The RE-TTA-*fcu* has fluorine on the linker as well as in the cluster. The MOFs were characterized by powder X-ray diffraction, X-ray photoelectron spectroscopy, thermogravimetric analysis, and contact angle measurements.

## 1. Introduction

Hydrophobic materials, particularly hydrophobic metal–organic frameworks (MOFs), are desirable for many applications, such as carbon dioxide capture and water purification [1,2,3,4]. The hydrophobicity of the material depends on the surface energy of the material and these energies range from 6 millinewtons per meter (mN/m) to a few thousand mN/m [1]. The -CF_3_ groups are known to have the lowest surface energies of 6–7 mN/m for fully covered surfaces [5]. The exchange of one fluorine in the -CF_3_ groups almost doubles the surface energy to 15 mN/m. The methyl group (-CH_3_) has a free energy of 20 mN/m and is still considered a hydrophobic group. Generally, metals and metal oxides exhibit high surface energies, such as MgO having a surface energy of 1200 mN/m and silicon has a surface energy of 1240 mN/m [6]. Hence, the lowest surface energies are exhibited in the highly fluorinated surfaces. Decreasing the surface energy is the key to achieving high hydrophobicity in materials. In addition to hydrophobicity, organofluorine molecules are resistant to environmental oxidation [3].

**Figure 1:**
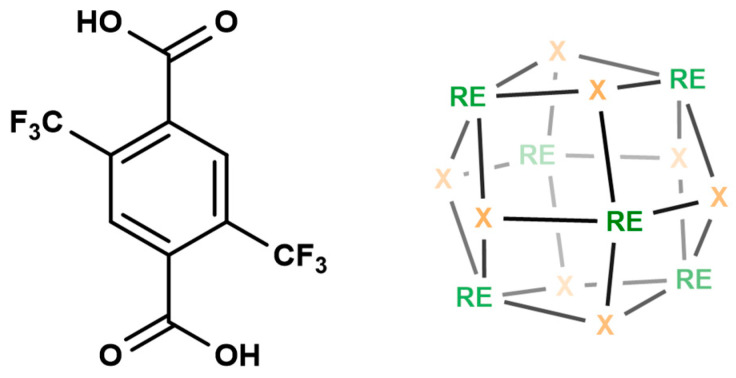
Structure of the fluorinated (left) organic linker 2,5-bis(trifluoromethyl)terephthalic acid (TTA) and (right) RE hexacluster (X = OH^−^ or F^−^).

Rare-earth metal–organic frameworks (RE-MOFs) are desirable due to their unique electronic, magnetic, and optical properties [7,8,9]. Rare-earth ions have high coordination numbers, ranging from 7 to 12 [9]. This high coordination number offers opportunities to prepare MOFs’ structures not observed in transition metals [10,11]. Many of the coordination sites can be occupied by the solvent molecules, and the removal of the solvent molecules may result in open metal sites [12]. These open metal sites can be utilized for selective guest molecule capture or sensing [13]. Recently, it was found that fluorinated triclusters, hexaclusters, or nonaclusters can be introduced in the RE-MOFs using organofluorine molecules in the synthesis [14,15,16,17]. In this work, highly fluorinated MOFs based on fluorinated RE hexaclusters and the fluorinated organic linker 2,5-bis(trifluoromethyl)terephthalic acid (TTA) were prepared (Figure 1). The organic linker, 2,5-bis(trifluoromethyl)terephthalic acid (TTA), has two hydrophobic -CF_3_ groups, making it a good candidate for fluorinated RE-MOFs. The hexacluster consists of six RE metal ions bridged by eight fluoride ions, having a formula of RE_6_X_8_.

In this work, two types of RE-MOFs (RE = Y, Gd, or Eu) have been prepared. The reaction of 2,5-bis(trifluoromethyl)terephthalic acid (TTA) with RE(III) ions resulted in an MOF with primitive cubic (*pcu*) topology named RE-TTA-*pcu* [18]. The RE-TTA-*pcu* is a three-dimensional microporous framework with binuclear RE nodes. The same reaction, in the presence of 2-fluorobenzoic acid, resulted in the UiO-66 analogue MOF with face-centered cubic (*fcu*) topology, named RE-TTA-*fcu* [14]. RE-TTA-*fcu* has metal hexaclusters bridged by hydroxy or fluoride ions (Figure 1).

## 2. Experimental

### Materials and Methods

The following materials were purchased from Fisher Scientific and Ambeed and used without further purification: europium(III) acetate hydrate (Eu(CH_3_CO_2_)_3_·6H_2_O, 99.9%), gadolinium(III) nitrate hexahydrate (Gd(NO_3_)_3_·6H_2_O, 99.9%), yttrium(III) nitrate hexahydrate (Y(NO_3_)_3_·6H_2_O, 99.9%), 2,5-bis(trifluoromethyl)terephthalic acid (TTA) (97%), nitric acid (ACS grade), N,N-dimethylformamide (ACS grade), 2-fluorobenzoic acid (97%). The ultra-high-purity gases (N_2_, CO_2_) for the gas adsorption analysis were purchased from Airgas.

Single-crystal X-ray diffraction data were collected on a D8-QUEST X-ray diffractometer (Bruker, Billerica, MA, USA) equipped with a Mo IμS microfocus X-ray source (λ = 0.71073 Å) at 200 K using an Oxford Cryosystems low-temperature device. Detailed crystallographic data are provided in Table 1 and Tables S1–S6. The X-ray photoelectron spectra were collected on a PHI VersaProbe II Scanning XPS Microprobe (Physical Electronics Inc, Chanhassen, MN, USA) equipped with Al Kα X-ray source (E_p_ = 1486.7 eV) at a pressure of 1.6 × 10^–9^ Torr. The high-resolution spectra were collected at the pass energy of 23.5 eV with a step size of 0.2 eV. The MOF samples were mounted on a double-sided copper tape and sputtered with metallic gold for charge reference. The data were processed with CasaXPS software (v 2.3) and binding energies were doubly calibrated to adventitious C_1s_ at 284.8 eV and Au 4f_7/2_ at 83.95 eV. A Bruker Avance III™ HD 600 MHz spectrometer (Bruker Biospin, Germany) was used to acquire the ^19^F NMR data at 298 K. The data were analyzed with TopSpin 4.1.0. for the ^19^F NMR spectra. A total of 10 mg of the MAF was digested in 20 µL solution of 10% D_2_SO_4_/D_2_O, and the mixture was added to 1 mL of DMSO-d_6_. The powder X-ray diffraction (PXRD) patterns were collected on an Ultima IV X-ray diffractometer (Rigaku, Tokyo, Japan) equipped with Cu K_α_ radiation, with a scan rate of 2°/min and a step size of 0.04°. The simulated XRD patterns were generated from the CIF files using the crystal structure visualization tool CCDC Mercury. SEM and EDX were performed on a Zeiss EVO LS SEM (Zeiss, Oberkochen, Germany) and an Aztec Instruments Oxford EDX. The water contact angles were measured using the Dataphysics Optical Contact Angle Measuring System (Future Digital Scientific Corp., New York, NY, USA). Thermogravimetric analysis was conducted using an SDT Q600 (TA Instruments New Castle, DE, USA). The samples were then heated from room temperature to 800 °C at a rate of 10 °C/min under air, with a flow rate of 20 mL/min. Detailed experimental procedures are provided in the supporting information.

The RE-TTA-*pcu* was prepared by dissolving Eu(III) acetate hydrate (34.2 mg, 0.104 mmol) and TTA (18 mg, 0.0596 mmol) in 11 mL of DMF. Concentrated nitric acid (0.05 mL) was added to the mixture and sonicated for two minutes. The reaction mixture was heated in a 20 mL glass vial for 24 h at 80 °C. Transparent colorless rectangular crystals were obtained. The crystals were washed with 5 mL of DMF (3x) and dried at 80 °C overnight. The yttrium and gadolinium MOFs were prepared using the same procedure by replacing the europium salt with the equivalent moles of the respective metal salts.

The RE-TTA-*fcu* was prepared by mixing Eu(III) acetate hydrate (34.2 mg, 0.104 mmol) and TTA (18 mg, 0.0596 mmol) in 11 mL of DMF. Then, 2-fluorobenzoic acid (240 mg, 1.71 mmol) was added to the reaction mixture. Concentrated nitric acid (0.10 mL) was added to lower the solution. The mixture was heated in a 20 mL glass vial at 120 °C for 24 h. Transparent colorless polygonal crystals were obtained, washed with 5 mL DMF (3x), and dried at 80 °C for 24 h. The yttrium and gadolinium MOFs were prepared using the same procedure by replacing the europium salt with the equivalent moles of the respective metal salts.

## 3. Results and Discussion

**Figure 2:**
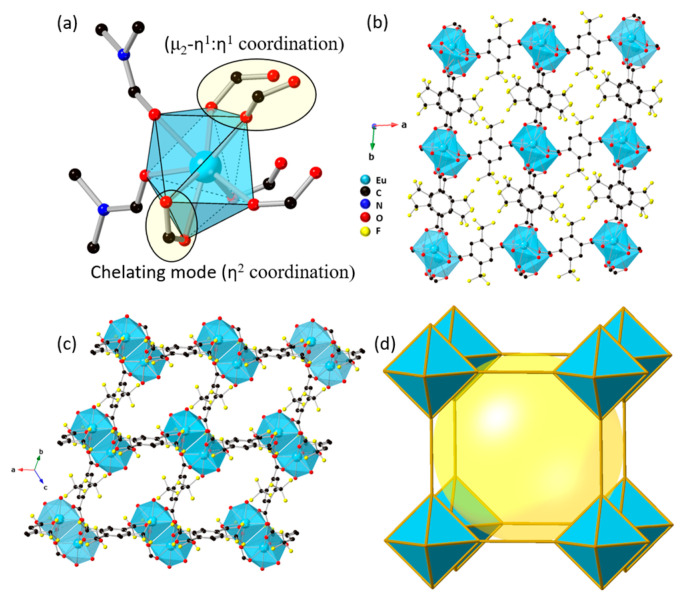
Crystal structure of the Eu-TTA-*pcu*: (**a**) asymmetric unit, (**b**) extended framework viewed along the c-direction, (**c**) extended framework viewed along the [111] lattice vector, and (**d**) the *pcu* topology of the framework, showing a central cavity with a diameter of 4.5 Å (yellow sphere).

Single-crystal X-ray diffraction analysis reveals that Eu-TTA-*pcu* is a three-dimensional framework, which crystallizes in the triclinic *P*$\bar{1}$ space group with a chemical formula {[Eu(TTA)_1.5_(DMF)]·(H_2_O)·(DMF)}_n_. The detailed crystallographic information is provided in Table 1. The asymmetric unit consists of one Eu(III) coordinated to two DMF molecules, which are two-fold disordered, three half TTA molecules, and a lattice water molecule. The TTA carboxylates exhibit η^2^ bidentate coordination modes. Two half TTA molecules bridge the neighboring Eu(III) ions via μ_2_-η^1^:η^1^ coordination of the carboxylate group to make a metal dimer node. The third TTA molecule exhibits a bidentate chelating coordination (η^2^) to a single Eu(III) ion and connects the consecutive dimer nodes. The Eu(III) ion exhibits a coordination number of nine. The RE-TTA-*pcu* is a three-dimensional framework with the primitive cubic (*pcu*) topology (Figure 2). The crystal structure packing reveals that the RE-TTA-*pcu* is a porous framework, having cavities with a diameter of 4.5 Å. The crystal packing shows that the ab-plane is densely packed, and the pores are completely blocked along the *c*-direction (Figure 2b).

**Figure 3:**
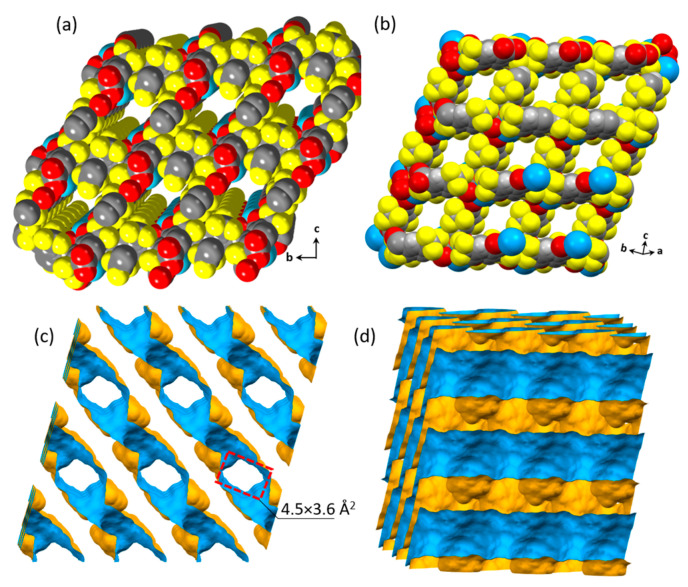
Space-filling model of the Eu-TTA-*pcu* (**a**) viewed along the a-axis and (**b**) viewed along the [111] vector. The channels and pore apertures (**c**) viewed along the a-axis, where channels are perpendicular to the plane, and (**d**) viewed along the c-axis, where channels are parallel to the plane. The inner surface of the channels is represented by the blue color.

The crystal packing of the RE-TTA-*pcu* shows that the framework is microporous with two-dimensional channels. These channels can be viewed along the a-direction and the [111] vector, as shown in Figure 3. The channels along the *a*-direction have a rectangular shape with the pore dimension of 4.5 × 3.6 Å^2^. The channels along the [111] vector are narrow, with the pore dimension of 2.6 × 2.8 Å^2^, making them inaccessible for gases such as nitrogen (d = 3.64 Å) and carbon dioxide (d = 3.34 Å). Theoretically, 41.3% of the unit cell volume is vacant if all the guest molecules are removed. However, gas adsorption analysis shows no uptake for both nitrogen and carbon dioxide. This is because of the coordinated DMF molecules, which are difficult to remove. The CO_2_ adsorption analysis shows that Gd-TTA-*fcu* is a porous structure with a Langmuir surface area of 58 m^2^/g (Figure S6). Nonetheless, RE-BDC-*fcu* MOFs have surface areas of up to 1200 m^2^/g [19,20].

**Figure 4:**
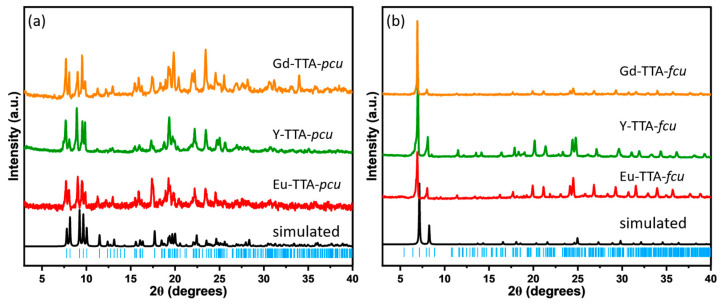
Powder XRD patterns of the (**a**) RE-TTA-pcu compared to the Eu-TTA-*pcu* simulated XRD pattern and (**b**) RE-TTA-*fcu* MOFs compared to the Ho-UiO-66 (CCDC 2080338).

The RE-TTA-*fcu* is a three-dimensional framework isostructural to the Ho-UiO-66 [14]. The powder XRD patterns of the RE-TTA-*fcu* are consistent with the simulated diffraction pattern for the Ho-UiO-66 (Figure 4b). This is due to the similar coordination geometry of the terephthalic acid and TTA. The linker TTA is a derivative of terephthalic acid with two (-CF_3_) groups present on the ortho positions to the carboxylate group. RE-TTA-*pcu* MOFs also exhibit a good match with the simulated XRD pattern from the Eu-TTA-*pcu* (Figure 4a). The good match with the simulated patterns confirms the phase purity of the RE-TTA-*pcu* MOFs.

Thermogravimetric analysis was carried out to analyze the thermal stabilities of the MOFs (Figure S1). It was found that all the MOFs exhibit thermal stability of up to 300 °C. The RE-TTA-*pcu* TGA curves exhibit a very small change of 2–3% when heated to 100 °C, showing a very small amount of water present in the pores. A second change of ~2 wt% after 150 °C was assigned to the loss of uncoordinated DMF molecules. A continuous mass loss after 170 °C indicates the loss of coordinated DMF molecules. A sudden mass loss was observed at ~320 °C, showing the decomposition of the MOFs. The TGA curves of the RE-TTA-*fcu* MOFs exhibited a 5–7 wt% loss on the initial heating up to 100 °C, indicating the presence of water molecules. A mass loss of 9 wt% after 150 °C was assigned to the DMF molecules. The RE-TTA-*fcu* MOFs decompose at ~325 °C and a higher residual mass was observed in comparison to the RE-TTA-*pcu*. The residual masses for RE-TTA-*pcu* ranged from 29–39 wt% in comparison to RE-TTA-*fcu* having 21–29 wt%. The high residual mass arises due to the presence of the metal hexaclusters.

**Figure 5:**
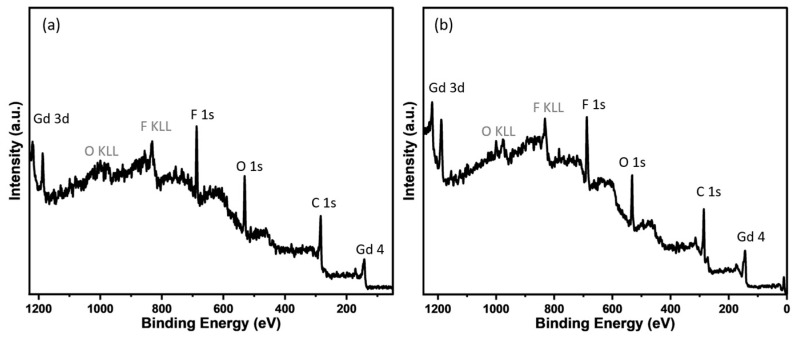
The XPS survey spectra of (**a**) Gd-TTA-*pcu*, and (**b**) Gd-TTA-*fcu*.

X-ray photoelectron spectroscopy analysis (XPS) was used to assess the elemental identities, chemical nature of the framework, and the purity of the MOFs. The survey spectra show the presence of Gd, C, O, and F. These chemical identities were also verified by the EDX spectrum (Figures S2–S4). The high-resolution spectra of the individual elements were obtained to assess the chemical nature of the framework (Figure 5).

**Figure 6:**
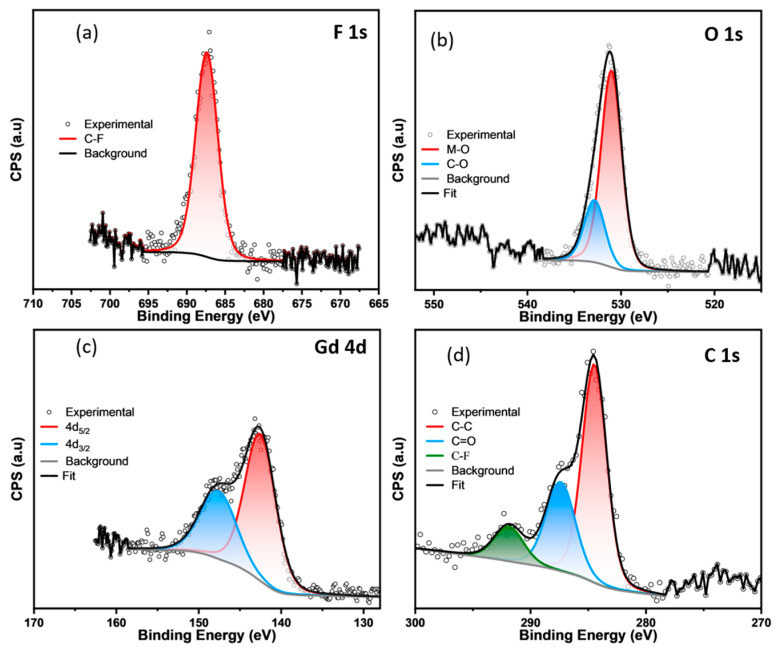
The high-resolution XPS spectra of the Gd-TTA-*pcu*: (**a**) F 1s (**b**) O 1s, (**c**) Gd 4d, and (**d**) C 1s.

The F 1s high-resolution XPS spectrum of the Gd-TTA-*pcu* shows that it has binding energy of 687.4 eV (Figure 6). The F 1s BE in the range of 686–690 are typical of the C-F species [17]. Therefore, this peak is assigned to the linker F in the -CF_3_ groups. No metal-fluorine species were found in the Gd-TTA-*pcu*. The F 1s in the metal fluorides shows BE in the range of 682–686 eV [21]. Moreover, this is cross-verified from the C 1s binding energy by the presence of the peak at 291.8 eV, which corresponds to fluorine bound carbon (Figure 6) [22]. The C 1s peaks at 284.4 and 287.4 eV are assigned to aromatic C=C and C-O species [23]. These observations are in agreement with the crystal structure. The high-resolution O 1s spectrum exhibits two peaks with binding energies 530.9 and 532.8, which are assigned to the carboxylate groups and DMF oxygen atoms. The Gd 4d spectrum shows a spin-orbital doublet 4d_5/2_ and 4d_3/2_ with binding energies of 142.5 and 147.6 eV [24]. These binding energies correspond to the Gd(III) species similar to Gd_2_O_3_ [24].

**Figure 7:**
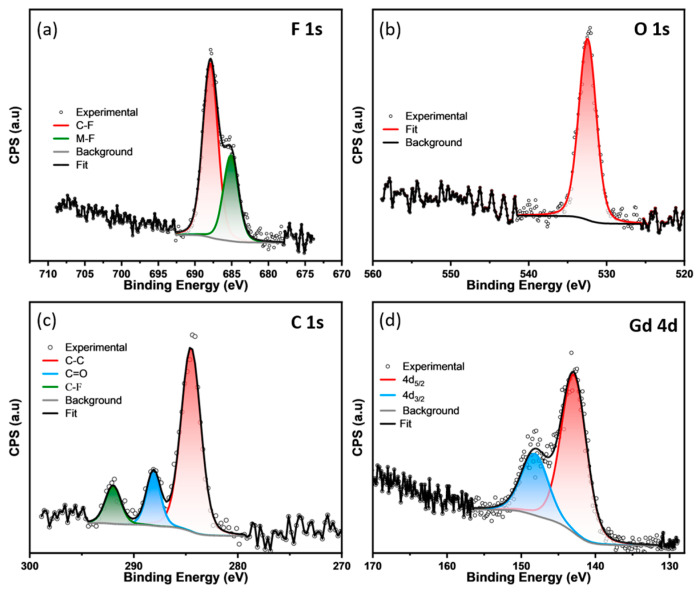
The high-resolution XPS spectra of the Gd-TTA-*fcu*: (**a**) F 1s (**b**) O 1s, (**c**) C 1s, and (**d**) Gd 4d.

The chemical nature of the Gd-TTA-*fcu* framework was analyzed using the high-resolution XPS spectra of the elements present in the MOF (Figure 7). The high-resolution scan of the F 1s in the Gd-TTA-*fcu* shows two distinct peaks, indicating the presence of two different chemical states of the fluorine. The peak with the lower binding energy at 685.1 eV is assigned to the fluorine present in the metal clusters. This binding energy is in the range of metal-fluorides [9]. The F 1s peak at the higher binding energy of 687.8 is assigned to the fluorine in the organic linker present as -CF_3_ groups, which is also confirmed from the C 1s spectrum by the presence of the C-F peak at 291.8 eV [22]. The ^19^F-NMR of the Gd-TTA-*fcu* also confirms the presence of fluorinated metal clusters (Figure S5). The acid-digested MOF in the D_2_SO_4_ exhibits the HF peak at −168.72 produced from the decomposition of the fluorinated cluster. The high-resolution O 1s spectrum shows a singlet peak at the binding energy of 532 eV, corresponding to the metal-bound carboxylate groups. The absence of low binding energy peaks around 530–531 eV indicates there are no bridging hydroxy groups. The C 1s spectra show peaks at 284.4 and 288.0, and 291.8 eV, which correspond to C=C, C=O, and C-F bonds in the organic linker. The Gd 4d spectrum shows a spin-orbital doublet 4d_5/2_ and 4d_3/2_ with binding energies of 142.8 and 148.0 eV [24].

**Figure 8:**
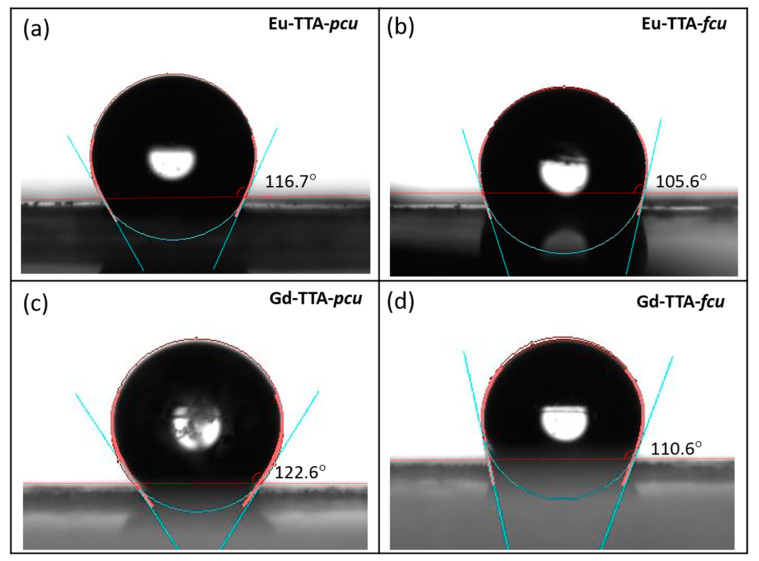
Contact angle measurement images of water on the surface of the RE-MOFs: (**a**) Eu-TTA-*pcu*, (**b**) Eu-TTA-*fcu*, (**c**) Gd-TTA-*pcu* and (**d**) Gd-TTA-*fcu*.

**Figure 9:**
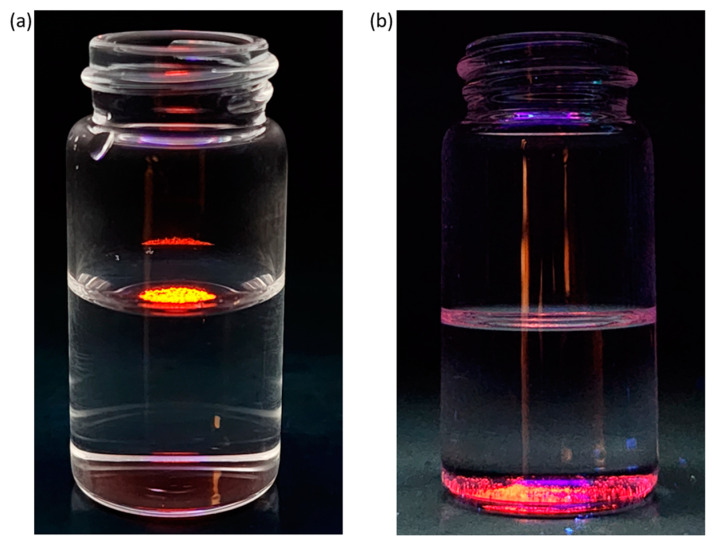
The Eu-TTA-*pcu* crystals (**a**) floating on water and (**b**) sinking in cyclohexane. The UV light (298 nm) was used for contrast.

The hydrophilicity/hydrophobicity of the MOFs were analyzed by water contact angle measurements (Figure 8). Materials with a water contact angle higher than 90 degrees are considered hydrophobic. It was found that the Eu-TTA-*pcu* exhibits a water contact angle of 16.7°. The Eu-TTA-*fcu*, on the other hand, exhibits a slightly lower contact angle of 105.6°. The Gd-TTA-*pcu* and Gd-TTA-*fcu* exhibit contact angles of 122.6° and 110.6°. The slight changes in the RE-TTA-*fcu* MOFs are attributed to the presence of additional fluorine in the metal clusters. Despite the presence of fluorine, the MOFs are not hydrophobic. This is possibly due to the presence of open metal sites in the MOFs, which may have coordinated water molecules. Additionally, the RE-TTA-*fcu* may have dimethyl ammonium (DMA) ions in the MOFs similar to the Ho-UiO-66 [14]. These DMA cations are generated by the breakdown of the dimethylformamide in solvothermal conditions [25,26,27]. Therefore, the hydrophobicity is decreased in the hexacluster-based MOFs. The hydrophobic nature of the RE-TTA-*pcu* was further demonstrated by adding crystals in non-polar and polar solvents (Figure 9). Since the crystals were colorless, a UV light (298 nm) was used for the fluorescence contrast against a dark background. The RE-TTA-*pcu* crystals did not sink in the water. However, in the non-polar solvent, cyclohexane, the crystals immediately sedimented. It is anticipated that the increase in hydrophobicity can be further increased by using more fluorinated linkers. This tunable nature makes these materials attractive for separation applications where gas molecules have small differences in their hydrophobicity.

## 4. Conclusions

Two new fluorinated hydrophobic rare-earth MOFs were synthesized using the fluorinated linker bis(trifluoromethyl)terephthalic acid and RE (RE: Y, Gd, or Eu) ions. In the absence of 2-fluorobenzoic acid, RE-TTA-*pcu* MOFs were obtained with fluorine present only on the linker. The crystal structure of the RE-TTA-*pcu* showed that they possessed binuclear rare-earth metal nodes and two-dimensional pore channels. The use of 2-fluorobenzoic acid resulted in MOFs with the *fcu* topology and fluorinated hexaclusters similar to RE-UiO-66. RE-TTA-*fcu* MOFs have fluorine on the linker as well as in the metal clusters. The MOFs were characterized via single-crystal XRD, powder XRD, ^19^F-NMR, EDS, XPS, and TGA analysis. The hydrophobicity of the MOFs was characterized by water contact angle measurements. The incorporation of fluorine in the metal clusters as well as on the linker holds may result in the development of hydrophobic MOFs for various applications, such as selective CO_2_ capture.

## Figures and Tables

**Table 1:** Crystallographic data of the Eu-TTA-*pcu*.

Name	Eu-TTA-*pcu*
Formula	{[Eu(TTA)_1.5_(DMF)]·(H_2_O)·(DMF)}_n_
Temperature/K	200
Space group	$P\bar{1}$
Crystal system	Triclinic
*a*/Å	10.0620(11)
*b*/Å	11.2464(12)
c/Å	12.3211(14)
*α* (°)	103.880(4)
*β* (°)	106.931(4)
*γ* (°)	92.980(4)
Volume (Å^3^)	1283.7(2)
Crystall Size (mm^3^)	0.100 × 0.080 × 0.070
2*θ* range for data collection/°	2.654 to 25.111°
Completeness to theta	99.7%
Reflection collected	25,679
Independent reflections	4563 [R(int) = 0.0433]
Goodness-of-fit on F^2^	1.197
Final *R* indexes[I ≥ 2σ(I)]	R_1_ = 0.0350, wR_2_ = 0.0858
Final *R* indexes [All Data]	*R*_1_ = 0.0422, w*R*_2_ = 0.0954

## Data Availability

Data are contained within the article. Additional data are provided in the supporting information file. CCDC 2362361 contains the supplementary crystallographic data for this paper. These data can be obtained free of charge from the Cambridge Crystallographic Data Centre via www.ccdc.cam.ac.uk.

## Supplementary Materials

The following supporting information can be downloaded at: https://www.mdpi.com/article/10.3390/ma17174213/s1.
