# Supplementary material for: Synthesis and Characterization of Highly Fluorinated Hydrophobic Rare–Earth Metal–Organic Frameworks (MOFs)
Source: Materials (Basel). 2024 Aug 26;17(17):4213. doi: 10.3390/ma17174213 (PMC11396249; doi:10.3390/ma17174213)
Supplement: Supplementary file 1 [file materials-17-04213-s001.zip › materials-3087114-supplementary.pdf]

materials

MDPI

Supplementary Materials

Synthesis and Characterization of Highly Fluorinated Hydrophobic Rare–Earth Metal–Organic Frameworks (MOFs)

Muhammad Abbas, Bhargavasairam Murari, Simin Sheybani, Monu Joy and Kenneth J. Balkus, Jr. *

Department of Chemistry and Biochemistry, The University of Texas at Dallas, 800 West Campbell Rd, Richardson, TX 75080, USA
* Correspondence: balkus@utdallas.edu

Citation: Abbas, M.; Murari, B.; Sheybani, S.; Joy, M.; Balkus, K.J., Jr. Synthesis and Characterization of Highly Fluorinated Hydrophobic Rare-Earth Metal–Organic Frameworks (MOFs). *Materials* **2024**, *17*, 4213. https://doi.org/10.3390/ma17174213

Academic Editor: Katsuhiko Ariga

Received: 17 June 2024
Revised: 15 August 2024
Accepted: 23 August 2024
Published: 26 August 2024

Copyright: © 2024 by the authors. Licensee MDPI, Basel, Switzerland. This article is an open access article distributed under the terms and conditions of the Creative Commons Attribution (CC BY) license (https://creativecommons.org/licenses/by/4.0/).

Experimental

Single crystal X-ray diffraction (SC-XRD)

Single crystal X-ray diffraction data for the compound was collected on a Bruker D8-QUEST X-ray diffractometer equipped with a Mo IμS microfocus X-ray source ($\lambda = 0.71073$ Å) and a PHOTON II CPAD detector. The measurement was performed at 200 K using an Oxford Cryosystems low-temperature device. The APEX-IV software suite was used for data collection, cell refinement, and data reduction. Absorption corrections were applied using SADABS.[1] Space group assignments were determined by examination of systematic absences, E-statistics, and successive refinement of the structures. The structure was solved with the dual-space method using SHELXT[2] and refined against $|F|^2$ on all data by full-matrix least squares with SHELXL.[3] All non-hydrogen atoms were refined with anisotropic displacement parameters. CCDC 2362361 contains the supplementary crystallographic data for this paper. Detailed crystallographic data is provided in Table 1 and Table S1-6. These data can be obtained free of charge from the Cambridge Crystallographic Data Centre via www.ccdc.cam.ac.uk.

X-ray Photoelectron Spectroscopy (XPS)

X-ray photoelectron spectra were collected on a PHI VersaProbe II Scanning XPS Microprobe (Physical Electronics Inc, Chanhassen, Minnesota) equipped with Al Kα X-ray source (E_p =1486.7 eV) at a pressure 1.6×10^{-9} Torr. The high-resolution spectra were collected at the pass energy of 23.5 eV with a step size of 0.2 eV. Photoelectron spectra were obtained using a charge compensation of 2 μA. The sample surface was cleaned by sputtering Gas-Cluster Ion Beam (GCIB) with an energy of 5 kV and cluster size of ~2500 argon atoms. The data was processed with software CasaXPS and binding energies were doubly calibrated to adventitious C_{1s} at 284.8 eV and Au $4f_{7/2}$ at 83.95 eV.

Nuclear Magnetic Resonance (NMR)

Bruker Avance III™ HD 600 MHz spectrometer was used to acquire ^{19}F NMR data at 298 K. The data was analyzed with TopSpin 4.1.0. for the ^{19}F NMR spectra. 10 mg of the MAF was digested in 20 μL solution of 10% D_2SO_4/D_2O, the mixture was added to 1 mL of DMSO-d_6.

Powder X-ray diffraction (PXRD)

PXRD patterns were collected on an Ultima IV X-ray diffractometer (Rigaku) equipped with Cu K_α radiation, with a scan rate of 2°/min and a step size of 0.04°. The simulated XRD patterns were generated from the CIF files using a crystal structure visualization tool Mercury[9] (Cambridge Crystallographic Data Centre).

The scanning electron microscopy (SEM) and Energy Dispersive X-ray Spectroscopy (EDX)

SEM and EDX was performed on a Ziess EVO LS SEM and an Aztec Instruments Oxford EDX

Thermogravimetric analysis (TGA)

Thermogravimetric analysis was conducted using a SDT Q600 (TA Instruments). The samples were then heated from room temperature to 800 °C at a rate of 10 °C/min under air with a flow rate of 20 mL/min.

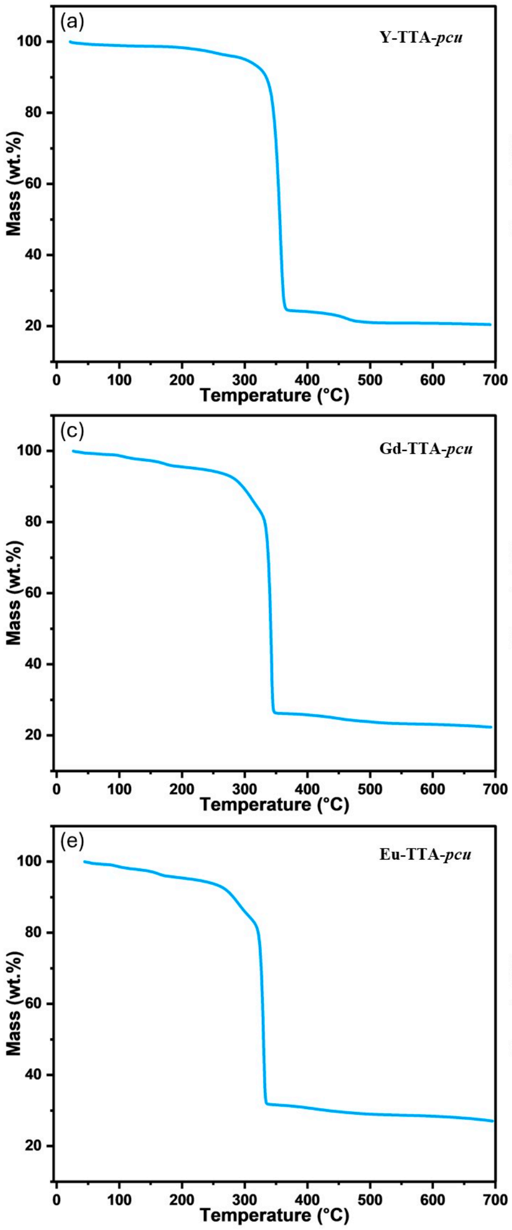

Figure S1. Thermogravimetric curves of the (a) Y-TTA-*pcu*, (b) Y-TTA-*fcu*, (c) Gd-TTA-*pcu*, (d) Gd-TTA-*fcu*, (e) Eu-TTA-*pcu*, and (f) Eu-TTA-*fcu*.

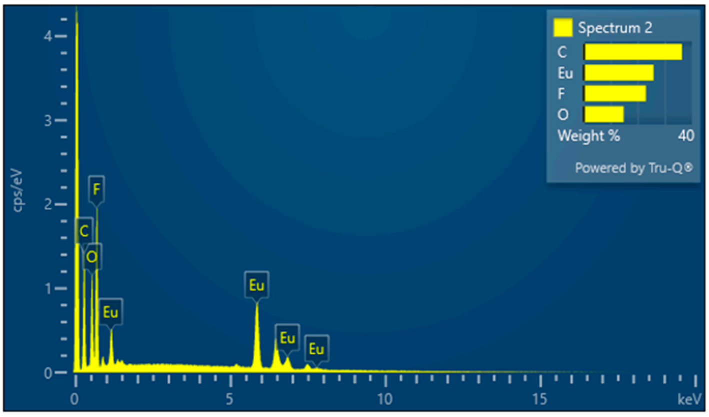

Element	Calculated	Exp. 1	Exp. 2	Average
Carbon	33.43364	36.3	39.53	37.915 ± 1.61
Oxygen	14.84609	14.82	14.93	14.875 ± 0.05
Fluorine	22.66584	23.02	21.88	22.45 ± 0.57
Europium	20.14423	25.86	23.66	24.76 ± 1.10

Figure S2. EDX spectra of the Eu-TTA-*pcu*. Elemental composition calculated using the chemical formula obtained from the Eu-TTA-*pcu* and compared to the average elemental weight% from the experimental EDS spectra. Calculated values were obtained from the Eu-TTA-*pcu* crystal structure including the solvent molecules. Excess carbon arises from the carbon tape.

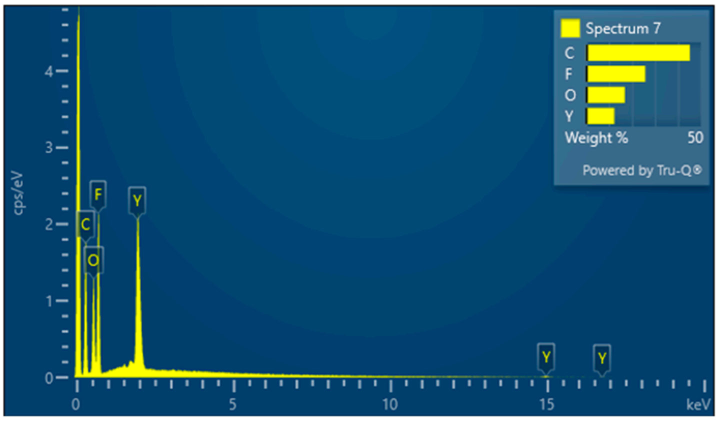

Element	Calculated	Exp. 1	Exp. 2	Average
Carbon	36.48344	45.17	50.55	47.86 ± 1.12
Oxygen	16.20035	16.83	17.55	17.19 ± 0.05
Fluorine	24.7334	25.74	22.72	24.23 ± 0.46
Yttrium	12.85983	12.26	9.18	10.72 ± 0.71

Figure S3. EDX spectra of the Y-TTA-*pcu*. Elemental composition calculated using the chemical formula obtained from the Eu-TTA-*pcu* by replacing the metal with yttrium and compared to the average elemental weight% from the experimental EDS spectra. Calculated values were obtained from the Eu-TTA-*pcu* crystal structure (by replacing the metal with yttrium) including the solvent molecules. Excess carbon arises from the carbon tape and possibly variation in coordinated and guest solvent molecules.

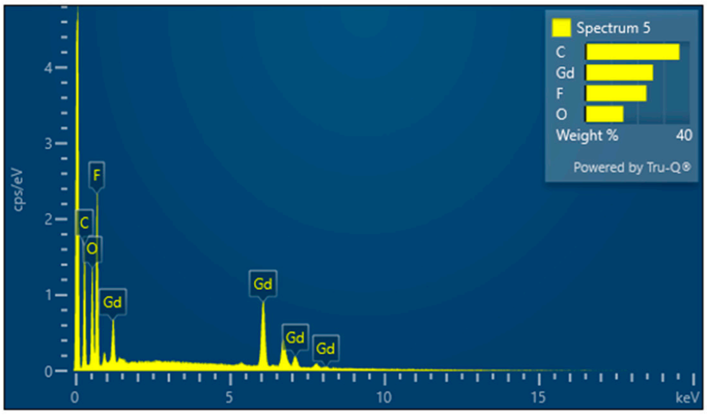

Element	Calculated	Exp. 1	Exp. 2	Average
Carbon	33.20082	35.99	40.48	38.23 ± 1.12
Oxygen	14.74271	14.66	14.88	14.77 ± 0.05
Fluorine	22.508	23.41	21.55	22.48 ± 0.46
Gadolinium	20.70032	25.94	23.1	24.52 ± 0.71

Figure S4. EDX spectra of the Eu-TTA-*pcu*. Elemental composition calculated using the chemical formula obtained from the Eu-TTA-*pcu* by replacing the metal with gadolinium and compared to the average elemental weight% from the experimental EDS spectra. Calculated values were obtained from the Eu-TTA-*pcu* crystal structure (by replacing the metal with gadolinium) including the solvent molecules. Excess carbon arises from the carbon tape and possibly variation in coordinated and guest solvent molecules.

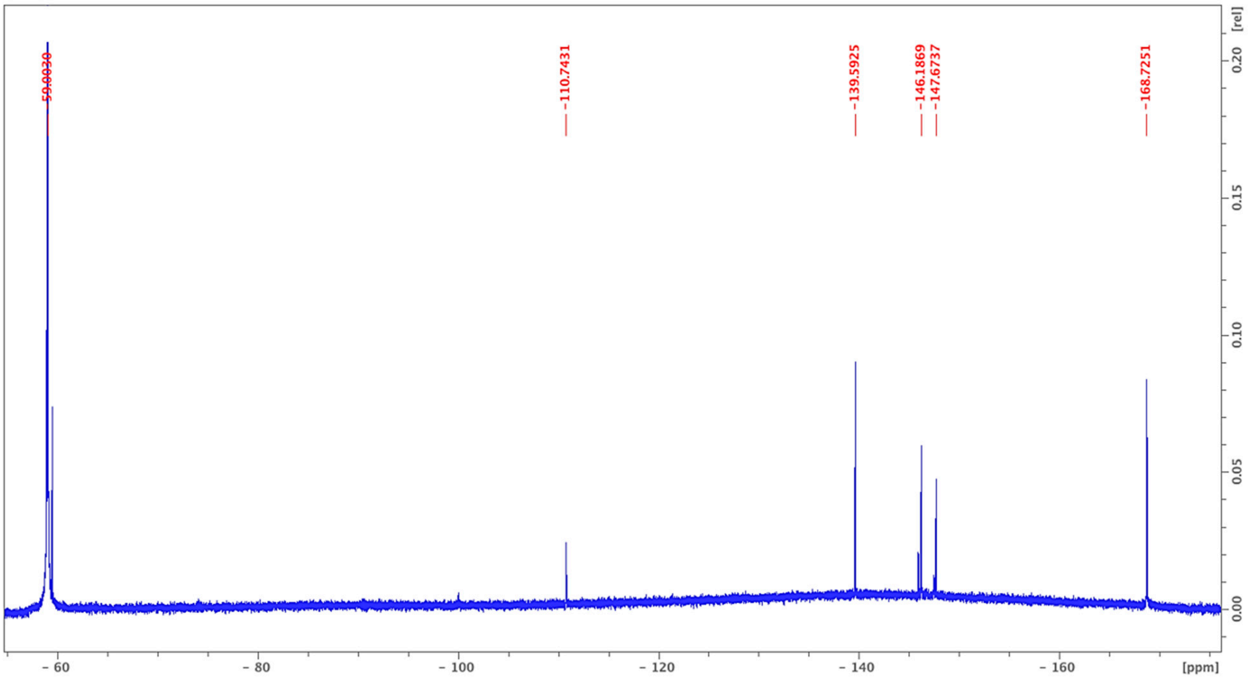

Figure S5. The ^{19}F-NMR of the Gd-TTA-*fcu* digested in D_2SO_4 exhibiting the HF peak at -168.72 produced from the decomposition of fluorinated cluster. The peaks at -59 and -139.5 are assigned to the linker CF_3 group. The presence of -110.7 peak indicates the presence of a small amount of 2-FBA.

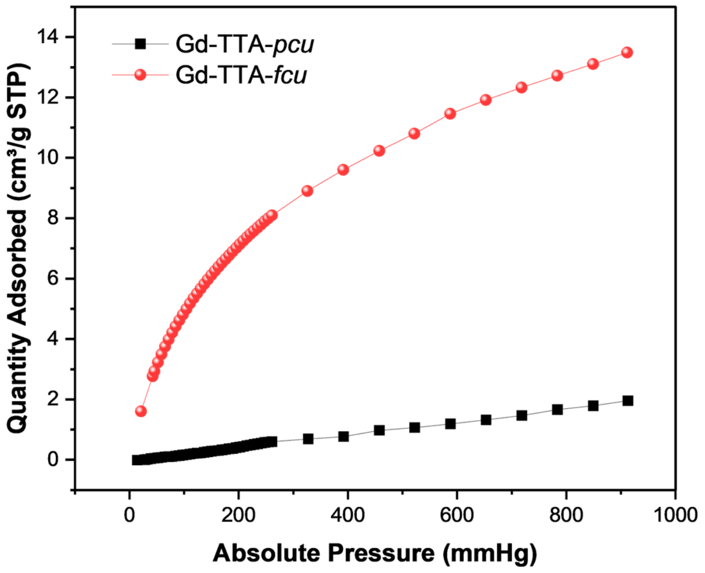

Figure S6. CO_2 adsorption isotherms at 273 K for Gd-TTA-*pcu* and Gd-TTA-*fcu*.

Table S1. Atomic coordinates (x 10^4) and equivalent isotropic displacement parameters (Å^{2}x 10^3) for Eu-TTA-*pcu*. U(eq) is defined as one third of the trace of the orthogonalized U^{ij} tensor.

	x	y	z	U(eq)
Eu(01)	5272(1)	5475(1)	6770(1)	19(1)
F(1)	2372(14)	10474(12)	11536(10)	41(2)

F(2)	1567(12)	10987(13)	9906(13)	47(2)
F(3A)	1520(11)	9084(8)	9921(13)	49(3)
F(4)	-516(4)	2702(4)	2222(3)	51(1)
F(5)	-1102(4)	1812(3)	3399(4)	54(1)
F(6)	1058(4)	2177(3)	3552(4)	43(1)
F(7)	8126(4)	2265(4)	6179(4)	52(1)
F(8)	7477(4)	2068(4)	7651(3)	53(1)
F(9)	8572(4)	700(4)	6867(4)	54(1)
O(1)	4082(4)	6944(4)	7892(4)	34(1)
O(2)	6332(4)	7418(4)	8254(4)	36(1)
O(3)	3556(4)	4699(4)	4866(3)	27(1)
O(4)	2187(4)	4357(4)	3042(3)	29(1)
O(5)	5624(4)	3368(3)	6080(3)	26(1)
O(6)	5191(6)	2844(4)	4122(4)	46(1)
O(7)	6442(4)	4981(4)	8596(3)	36(1)
O(8)	3506(4)	4165(4)	7106(4)	36(1)
O(9)	4180(14)	3575(13)	9688(11)	36(3)
N(1A)	8200(50)	5260(40)	10340(30)	40(3)
N(2)	2995(6)	2188(5)	7121(5)	42(1)
C(1)	5192(6)	7678(5)	8397(4)	24(1)
C(2)	5144(6)	8909(5)	9218(4)	25(1)
C(3)	3887(6)	9096(5)	9432(5)	27(1)
C(4)	3706(6)	10163(5)	10194(5)	29(1)
C(5A)	2303(10)	10192(10)	10412(11)	37(2)
C(6)	2351(5)	4576(5)	4118(4)	22(1)
C(7)	1125(5)	4759(5)	4568(4)	22(1)
C(8)	-69(5)	3880(5)	4189(4)	22(1)
C(9)	-1178(5)	4138(5)	4612(5)	24(1)
C(10)	-154(6)	2649(5)	3335(5)	31(1)
C(11)	5373(6)	2614(5)	5086(5)	28(1)
C(12)	5250(6)	1259(5)	5058(5)	24(1)
C(13)	6223(6)	722(5)	5783(5)	25(1)
C(14)	5968(6)	-519(5)	5729(5)	26(1)
C(15)	7598(7)	1442(5)	6617(6)	37(1)
C(16)	7535(6)	5540(6)	9364(5)	35(1)
C(17A)	9370(30)	6120(30)	11250(30)	51(5)
C(18A)	7780(40)	4080(20)	10520(20)	53(5)
C(19A)	3822(12)	3202(10)	7434(10)	41(2)

C(19B)	2834(16)	3146(12)	6660(13)	42(2)
C(21A)	1651(13)	1947(13)	6215(12)	56(3)
C(21B)	4100(20)	2407(19)	8275(16)	67(4)
C(20A)	3424(16)	1049(12)	7487(14)	63(3)
C(20B)	2180(20)	1033(15)	6615(19)	65(4)
F(1A)	2010(20)	10380(20)	11220(20)	47(3)
C(5B)	2210(15)	10324(18)	10180(20)	39(3)
F(3B)	1275(15)	9353(17)	9430(20)	48(4)
F(2A)	1760(20)	11329(19)	9850(20)	42(4)
N(1B)	8060(40)	5200(30)	10330(20)	40(3)
C(18B)	7290(30)	4218(18)	10573(14)	47(4)
C(17B)	9400(30)	5810(30)	11210(20)	53(5)

Table S2. Bond lengths [Å] and angles [°] for Eu-TTA-*pcu*.

Eu(01)-O(3)	2.397(4)
Eu(01)-O(5)	2.400(3)
Eu(01)-O(6)#1	2.406(4)
Eu(01)-O(7)	2.426(4)
Eu(01)-O(8)	2.438(4)
Eu(01)-O(2)	2.442(4)
Eu(01)-O(1)	2.480(4)
Eu(01)-O(4)#1	2.492(4)
Eu(01)-O(3)#1	2.590(4)
F(1)-C(5A)	1.325(11)
F(2)-C(5A)	1.343(12)
F(3A)-C(5A)	1.341(10)
F(4)-C(10)	1.329(7)
F(5)-C(10)	1.338(7)
F(6)-C(10)	1.336(7)
F(7)-C(15)	1.335(7)
F(8)-C(15)	1.343(8)
F(9)-C(15)	1.337(7)
O(1)-C(1)	1.253(7)
O(2)-C(1)	1.249(7)
O(3)-C(6)	1.268(6)
O(4)-C(6)	1.248(6)
O(5)-C(11)	1.258(6)

O(6)-C(11)	1.240(7)
O(7)-C(16)	1.240(7)
O(8)-C(19B)	1.214(13)
O(8)-C(19A)	1.269(11)
O(9)-H(9A)	0.8697
O(9)-H(9B)	0.8691
N(1A)-C(16)	1.313(14)
N(1A)-C(18A)	1.456(18)
N(1A)-C(17A)	1.481(17)
N(2)-C(19A)	1.282(11)
N(2)-C(19B)	1.329(13)
N(2)-C(20B)	1.398(15)
N(2)-C(21A)	1.444(13)
N(2)-C(21B)	1.486(15)
N(2)-C(20A)	1.501(13)
C(1)-C(2)	1.518(7)
C(2)-C(3)	1.381(8)
C(2)-C(4)#2	1.406(8)
C(3)-C(4)	1.392(7)
C(3)-H(3)	0.9500
C(4)-C(5A)	1.513(11)
C(4)-C(5B)	1.522(15)
C(6)-C(7)	1.498(7)
C(7)-C(9)#3	1.386(7)
C(7)-C(8)	1.402(7)
C(8)-C(9)	1.379(7)
C(8)-C(10)	1.506(7)
C(9)-H(9)	0.9500
C(11)-C(12)	1.514(7)
C(12)-C(13)	1.389(8)
C(12)-C(14)#4	1.396(8)
C(13)-C(14)	1.387(7)
C(13)-C(15)	1.511(8)
C(14)-H(14)	0.9500
C(16)-N(1B)	1.309(12)
C(16)-H(16)	0.9500
C(16)-H(16A)	0.9500
C(17A)-H(17A)	0.9800

C(17A)-H(17B)	0.9800
C(17A)-H(17C)	0.9800
C(18A)-H(18A)	0.9800
C(18A)-H(18B)	0.9800
C(18A)-H(18C)	0.9800
C(19A)-H(19A)	0.9500
C(19B)-H(19B)	0.9500
C(21A)-H(21A)	0.9800
C(21A)-H(21B)	0.9800
C(21A)-H(21C)	0.9800
C(21B)-H(21D)	0.9800
C(21B)-H(21E)	0.9800
C(21B)-H(21F)	0.9800
C(20A)-H(20A)	0.9800
C(20A)-H(20B)	0.9800
C(20A)-H(20C)	0.9800
C(20B)-H(20D)	0.9800
C(20B)-H(20E)	0.9800
C(20B)-H(20F)	0.9800
F(1A)-C(5B)	1.336(16)
C(5B)-F(2A)	1.348(17)
C(5B)-F(3B)	1.355(15)
N(1B)-C(18B)	1.450(16)
N(1B)-C(17B)	1.475(14)
C(18B)-H(18D)	0.9800
C(18B)-H(18E)	0.9800
C(18B)-H(18F)	0.9800
C(17B)-H(17D)	0.9800
C(17B)-H(17E)	0.9800
C(17B)-H(17F)	0.9800

O(3)-Eu(01)-O(5)	74.32(13)
O(3)-Eu(01)-O(6)#1	71.36(14)
O(5)-Eu(01)-O(6)#1	134.50(13)
O(3)-Eu(01)-O(7)	144.42(13)
O(5)-Eu(01)-O(7)	78.08(13)
O(6)#1-Eu(01)-O(7)	143.39(14)
O(3)-Eu(01)-O(8)	75.96(14)

O(5)-Eu(01)-O(8)	72.39(14)
O(6)#1-Eu(01)-O(8)	124.97(17)
O(7)-Eu(01)-O(8)	74.59(14)
O(3)-Eu(01)-O(2)	140.73(14)
O(5)-Eu(01)-O(2)	143.39(13)
O(6)#1-Eu(01)-O(2)	71.00(15)
O(7)-Eu(01)-O(2)	72.40(15)
O(8)-Eu(01)-O(2)	118.32(15)
O(3)-Eu(01)-O(1)	103.89(13)
O(5)-Eu(01)-O(1)	147.30(13)
O(6)#1-Eu(01)-O(1)	71.08(15)
O(7)-Eu(01)-O(1)	87.61(15)
O(8)-Eu(01)-O(1)	75.52(14)
O(2)-Eu(01)-O(1)	52.74(13)
O(3)-Eu(01)-O(4)#1	119.84(12)
O(5)-Eu(01)-O(4)#1	76.95(13)
O(6)#1-Eu(01)-O(4)#1	95.17(16)
O(7)-Eu(01)-O(4)#1	74.03(14)
O(8)-Eu(01)-O(4)#1	139.73(14)
O(2)-Eu(01)-O(4)#1	74.51(13)
O(1)-Eu(01)-O(4)#1	127.23(12)
O(3)-Eu(01)-O(3)#1	69.66(13)
O(5)-Eu(01)-O(3)#1	70.69(12)
O(6)#1-Eu(01)-O(3)#1	69.91(14)
O(7)-Eu(01)-O(3)#1	121.02(13)
O(8)-Eu(01)-O(3)#1	134.77(13)
O(2)-Eu(01)-O(3)#1	106.89(13)
O(1)-Eu(01)-O(3)#1	140.39(14)
O(4)#1-Eu(01)-O(3)#1	51.16(12)
C(1)-O(1)-Eu(01)	91.7(3)
C(1)-O(2)-Eu(01)	93.5(3)
C(6)-O(3)-Eu(01)	154.5(3)
C(6)-O(3)-Eu(01)#1	91.0(3)
Eu(01)-O(3)-Eu(01)#1	110.35(13)
C(6)-O(4)-Eu(01)#1	96.1(3)
C(11)-O(5)-Eu(01)	135.1(3)
C(11)-O(6)-Eu(01)#1	140.2(4)
C(16)-O(7)-Eu(01)	127.0(4)

C(19B)-O(8)-Eu(01)	138.6(8)
C(19A)-O(8)-Eu(01)	119.9(5)
H(9A)-O(9)-H(9B)	104.5
C(16)-N(1A)-C(18A)	119.6(17)
C(16)-N(1A)-C(17A)	120.5(18)
C(18A)-N(1A)-C(17A)	119.8(15)
C(19B)-N(2)-C(20B)	124.7(12)
C(19A)-N(2)-C(21A)	123.5(8)
C(19B)-N(2)-C(21B)	116.0(10)
C(20B)-N(2)-C(21B)	119.2(12)
C(19A)-N(2)-C(20A)	123.4(9)
C(21A)-N(2)-C(20A)	112.2(9)
O(2)-C(1)-O(1)	121.8(5)
O(2)-C(1)-C(2)	119.4(5)
O(1)-C(1)-C(2)	118.7(5)
C(3)-C(2)-C(4)#2	118.3(5)
C(3)-C(2)-C(1)	116.9(5)
C(4)#2-C(2)-C(1)	124.8(5)
C(2)-C(3)-C(4)	123.0(5)
C(2)-C(3)-H(3)	118.5
C(4)-C(3)-H(3)	118.5
C(3)-C(4)-C(2)#2	118.7(5)
C(3)-C(4)-C(5A)	116.9(6)
C(2)#2-C(4)-C(5A)	124.2(6)
C(3)-C(4)-C(5B)	116.5(8)
C(2)#2-C(4)-C(5B)	124.1(8)
F(1)-C(5A)-F(3A)	105.7(9)
F(1)-C(5A)-F(2)	108.4(9)
F(3A)-C(5A)-F(2)	106.0(9)
F(1)-C(5A)-C(4)	114.8(8)
F(3A)-C(5A)-C(4)	110.7(7)
F(2)-C(5A)-C(4)	110.8(9)
O(4)-C(6)-O(3)	121.6(5)
O(4)-C(6)-C(7)	120.5(5)
O(3)-C(6)-C(7)	117.9(4)
C(9)#3-C(7)-C(8)	119.0(5)
C(9)#3-C(7)-C(6)	117.8(5)
C(8)-C(7)-C(6)	123.2(5)

C(9)-C(8)-C(7)	119.7(5)
C(9)-C(8)-C(10)	119.3(5)
C(7)-C(8)-C(10)	121.0(5)
C(8)-C(9)-C(7)#3	121.3(5)
C(8)-C(9)-H(9)	119.3
C(7)#3-C(9)-H(9)	119.3
F(4)-C(10)-F(6)	107.0(5)
F(4)-C(10)-F(5)	106.8(5)
F(6)-C(10)-F(5)	106.0(5)
F(4)-C(10)-C(8)	112.9(5)
F(6)-C(10)-C(8)	112.5(5)
F(5)-C(10)-C(8)	111.1(5)
O(6)-C(11)-O(5)	128.0(5)
O(6)-C(11)-C(12)	115.9(5)
O(5)-C(11)-C(12)	116.1(5)
C(13)-C(12)-C(14)#4	118.5(5)
C(13)-C(12)-C(11)	124.9(5)
C(14)#4-C(12)-C(11)	116.5(5)
C(14)-C(13)-C(12)	120.2(5)
C(14)-C(13)-C(15)	118.0(5)
C(12)-C(13)-C(15)	121.8(5)
C(13)-C(14)-C(12)#4	121.3(5)
C(13)-C(14)-H(14)	119.4
C(12)#4-C(14)-H(14)	119.4
F(7)-C(15)-F(9)	106.7(5)
F(7)-C(15)-F(8)	106.9(5)
F(9)-C(15)-F(8)	106.3(5)
F(7)-C(15)-C(13)	112.7(5)
F(9)-C(15)-C(13)	111.6(5)
F(8)-C(15)-C(13)	112.3(5)
O(7)-C(16)-N(1A)	127.4(11)
O(7)-C(16)-N(1B)	121.5(9)
O(7)-C(16)-H(16)	116.3
N(1A)-C(16)-H(16)	116.3
O(7)-C(16)-H(16A)	119.2
N(1B)-C(16)-H(16A)	119.2
N(1A)-C(17A)-H(17A)	109.4
N(1A)-C(17A)-H(17B)	109.5

H(17A)-C(17A)-H(17B)	109.5
N(1A)-C(17A)-H(17C)	109.5
H(17A)-C(17A)-H(17C)	109.5
H(17B)-C(17A)-H(17C)	109.5
N(1A)-C(18A)-H(18A)	109.5
N(1A)-C(18A)-H(18B)	109.5
H(18A)-C(18A)-H(18B)	109.5
N(1A)-C(18A)-H(18C)	109.4
H(18A)-C(18A)-H(18C)	109.5
H(18B)-C(18A)-H(18C)	109.5
O(8)-C(19A)-N(2)	124.9(10)
O(8)-C(19A)-H(19A)	117.5
N(2)-C(19A)-H(19A)	117.5
O(8)-C(19B)-N(2)	125.5(12)
O(8)-C(19B)-H(19B)	117.3
N(2)-C(19B)-H(19B)	117.3
N(2)-C(21A)-H(21A)	109.5
N(2)-C(21A)-H(21B)	109.5
H(21A)-C(21A)-H(21B)	109.5
N(2)-C(21A)-H(21C)	109.5
H(21A)-C(21A)-H(21C)	109.5
H(21B)-C(21A)-H(21C)	109.5
N(2)-C(21B)-H(21D)	109.5
N(2)-C(21B)-H(21E)	109.5
H(21D)-C(21B)-H(21E)	109.5
N(2)-C(21B)-H(21F)	109.5
H(21D)-C(21B)-H(21F)	109.5
H(21E)-C(21B)-H(21F)	109.5
N(2)-C(20A)-H(20A)	109.5
N(2)-C(20A)-H(20B)	109.5
H(20A)-C(20A)-H(20B)	109.5
N(2)-C(20A)-H(20C)	109.5
H(20A)-C(20A)-H(20C)	109.5
H(20B)-C(20A)-H(20C)	109.5
N(2)-C(20B)-H(20D)	109.5
N(2)-C(20B)-H(20E)	109.5
H(20D)-C(20B)-H(20E)	109.5
N(2)-C(20B)-H(20F)	109.5

H(20D)-C(20B)-H(20F)	109.5
H(20E)-C(20B)-H(20F)	109.5
F(1A)-C(5B)-F(2A)	107.1(16)
F(1A)-C(5B)-F(3B)	103.8(15)
F(2A)-C(5B)-F(3B)	105.5(15)
F(1A)-C(5B)-C(4)	113.4(16)
F(2A)-C(5B)-C(4)	114.0(16)
F(3B)-C(5B)-C(4)	112.2(13)
C(16)-N(1B)-C(18B)	120.1(13)
C(16)-N(1B)-C(17B)	121.8(14)
C(18B)-N(1B)-C(17B)	118.0(13)
N(1B)-C(18B)-H(18D)	109.5
N(1B)-C(18B)-H(18E)	109.5
H(18D)-C(18B)-H(18E)	109.5
N(1B)-C(18B)-H(18F)	109.4
H(18D)-C(18B)-H(18F)	109.5
H(18E)-C(18B)-H(18F)	109.5
N(1B)-C(17B)-H(17D)	109.5
N(1B)-C(17B)-H(17E)	109.5
H(17D)-C(17B)-H(17E)	109.5
N(1B)-C(17B)-H(17F)	109.4
H(17D)-C(17B)-H(17F)	109.5
H(17E)-C(17B)-H(17F)	109.5

Symmetry transformations used to generate equivalent atoms:

#1 -x+1,-y+1,-z+1 #2 -x+1,-y+2,-z+2 #3 -x,-y+1,-z+1

#4 -x+1,-y,-z+1

Table S3. Anisotropic displacement parameters (Å^{2}x 10^3) for Eu-TTA-*pcu*. The anisotropic displacement factor exponent takes the form: -2p^2[h^{2}a*^{2}U^{11} + ... + 2 h k a* b* U^{12}]

	U^{11}	U^{22}	U^{33}	U^{23}	U^{13}	U^{12}
Eu(01)	23(1)	13(1)	19(1)	0(1)	8(1)	-1(1)
F(1)	43(5)	42(4)	37(4)	-6(3)	23(4)	-3(4)
F(2)	26(4)	51(5)	50(4)	-2(4)	8(3)	0(4)
F(3A)	39(4)	38(3)	60(5)	-17(3)	29(4)	-13(3)
F(4)	53(2)	51(2)	28(2)	-11(2)	2(2)	1(2)
F(5)	51(2)	24(2)	81(3)	-10(2)	33(2)	-13(2)

F(6)	36(2)	31(2)	57(2)	0(2)	14(2)	9(2)
F(7)	38(2)	37(2)	76(3)	20(2)	11(2)	-11(2)
F(8)	53(2)	43(2)	39(2)	-6(2)	-3(2)	-6(2)
F(9)	34(2)	40(2)	75(3)	17(2)	-1(2)	6(2)
O(1)	28(2)	26(2)	41(2)	-12(2)	19(2)	-7(2)
O(2)	26(2)	24(2)	43(2)	-15(2)	6(2)	0(2)
O(3)	16(2)	36(2)	27(2)	8(2)	4(1)	-5(2)
O(4)	18(2)	39(2)	25(2)	2(2)	6(1)	-1(2)
O(5)	40(2)	13(2)	24(2)	2(1)	11(2)	3(2)
O(6)	97(4)	17(2)	27(2)	7(2)	23(2)	9(2)
O(7)	41(2)	34(2)	25(2)	9(2)	2(2)	-7(2)
O(8)	35(2)	34(2)	45(2)	14(2)	21(2)	-5(2)
O(9)	40(6)	49(6)	37(6)	31(5)	20(5)	22(5)
N(1A)	39(6)	48(5)	27(4)	8(4)	4(4)	4(4)
N(2)	43(3)	41(3)	45(3)	21(2)	13(2)	-6(2)
C(1)	30(2)	24(2)	17(2)	2(2)	8(2)	0(2)
C(2)	33(3)	20(2)	19(2)	1(2)	8(2)	2(2)
C(3)	28(3)	20(2)	27(3)	-3(2)	7(2)	-1(2)
C(4)	30(2)	23(2)	30(3)	-4(2)	11(2)	0(2)
C(5A)	32(3)	33(4)	36(4)	-12(3)	13(3)	-7(3)
C(6)	21(2)	20(2)	23(2)	4(2)	7(2)	-2(2)
C(7)	20(2)	23(2)	23(2)	5(2)	7(2)	1(2)
C(8)	21(2)	22(2)	21(2)	1(2)	7(2)	-1(2)
C(9)	18(2)	25(3)	25(3)	3(2)	5(2)	-3(2)
C(10)	25(2)	27(3)	34(3)	-2(2)	8(2)	0(2)
C(11)	43(3)	15(2)	25(2)	3(2)	15(2)	2(2)
C(12)	34(3)	16(2)	24(2)	2(2)	16(2)	2(2)
C(13)	30(2)	20(2)	24(2)	3(2)	9(2)	1(2)
C(14)	33(3)	18(2)	25(3)	8(2)	7(2)	4(2)
C(15)	37(3)	22(3)	44(3)	4(2)	4(2)	-1(2)
C(16)	38(3)	32(3)	29(2)	7(2)	5(2)	-6(2)
C(17A)	35(7)	59(10)	41(7)	0(8)	-8(6)	15(8)
C(18A)	52(8)	62(6)	47(7)	27(5)	9(6)	9(6)
C(19A)	41(4)	41(4)	43(4)	17(3)	15(4)	-6(3)
C(19B)	44(5)	39(4)	43(5)	22(4)	10(4)	-12(4)
C(21A)	51(6)	52(6)	56(6)	13(5)	8(4)	-10(5)
C(21B)	70(8)	55(7)	64(7)	35(6)	-6(6)	-9(7)
C(20A)	74(7)	46(6)	69(7)	30(5)	14(6)	-7(5)

C(20B)	69(9)	47(6)	74(9)	23(7)	16(7)	-26(6)
F(1A)	37(7)	47(6)	47(6)	-11(6)	22(5)	-14(6)
C(5B)	30(4)	33(5)	40(5)	-13(4)	12(4)	-4(4)
F(3B)	36(5)	39(5)	52(6)	-17(5)	15(5)	-9(4)
F(2A)	24(7)	40(6)	44(6)	-11(6)	3(5)	0(6)
N(1B)	40(6)	49(5)	24(3)	7(3)	3(4)	0(4)
C(18B)	50(7)	61(6)	39(5)	26(4)	16(5)	9(5)
C(17B)	43(6)	61(10)	37(6)	-3(7)	-4(5)	9(7)

Table S4. Hydrogen coordinates (x 10^4) and isotropic displacement parameters (Å^{2}x 10^3) for Eu-TTA-*pcu*.

	x	y	z	U(eq)
H(9A)	3326	3733	9423	54
H(9B)	4599	3774	9219	54
H(3)	3110	8468	9040	33
H(9)	-1994	3549	4337	29
H(14)	6634	-871	6238	31
H(16)	7938	6255	9234	42
H(16A)	8003	6234	9251	42
H(17A)	9441	6909	11044	77
H(17B)	10252	5768	11296	77
H(17C)	9208	6267	12013	77
H(18A)	7002	3613	9829	80
H(18B)	7469	4229	11218	80
H(18C)	8571	3604	10626	80
H(19A)	4740	3234	7950	49
H(19B)	2133	3024	5920	50
H(21A)	1372	2726	6056	84
H(21B)	950	1563	6480	84
H(21C)	1720	1388	5494	84
H(21D)	4621	3236	8486	100
H(21E)	4751	1789	8221	100
H(21F)	3672	2341	8879	100
H(20A)	3534	436	6815	94
H(20B)	2702	705	7756	94
H(20C)	4315	1262	8129	94

H(20D)	1529	1022	5845	97
H(20E)	1647	875	7129	97
H(20F)	2790	391	6516	97
H(18D)	6289	4175	10170	71
H(18E)	7458	4392	11424	71
H(18F)	7612	3428	10293	71
H(17D)	9876	6355	10887	80
H(17E)	9999	5186	11422	80
H(17F)	9226	6300	11918	80

Table S5. Torsion angles [°] for Eu-TTA-*pcu*.

Eu(01)-O(2)-C(1)-O(1)	4.8(6)
Eu(01)-O(2)-C(1)-C(2)	-176.0(4)
Eu(01)-O(1)-C(1)-O(2)	-4.7(6)
Eu(01)-O(1)-C(1)-C(2)	176.1(4)
O(2)-C(1)-C(2)-C(3)	-173.9(5)
O(1)-C(1)-C(2)-C(3)	5.4(8)
O(2)-C(1)-C(2)-C(4)#2	3.3(8)
O(1)-C(1)-C(2)-C(4)#2	-177.4(5)
C(4)#2-C(2)-C(3)-C(4)	0.5(10)
C(1)-C(2)-C(3)-C(4)	177.9(5)
C(2)-C(3)-C(4)-C(2)#2	-0.5(10)
C(2)-C(3)-C(4)-C(5A)	-175.3(8)
C(2)-C(3)-C(4)-C(5B)	170.2(12)
C(3)-C(4)-C(5A)-F(1)	127.2(9)
C(2)#2-C(4)-C(5A)-F(1)	-47.3(14)
C(3)-C(4)-C(5A)-F(3A)	7.6(12)
C(2)#2-C(4)-C(5A)-F(3A)	-166.8(9)
C(3)-C(4)-C(5A)-F(2)	-109.7(9)
C(2)#2-C(4)-C(5A)-F(2)	75.9(12)
Eu(01)#1-O(4)-C(6)-O(3)	-5.1(5)
Eu(01)#1-O(4)-C(6)-C(7)	172.1(4)
Eu(01)-O(3)-C(6)-O(4)	152.5(6)
Eu(01)#1-O(3)-C(6)-O(4)	4.9(5)
Eu(01)-O(3)-C(6)-C(7)	-24.8(10)
Eu(01)#1-O(3)-C(6)-C(7)	-172.4(4)
O(4)-C(6)-C(7)-C(9)#3	-122.6(6)

O(3)-C(6)-C(7)-C(9)#3	54.8(7)
O(4)-C(6)-C(7)-C(8)	56.5(7)
O(3)-C(6)-C(7)-C(8)	-126.2(5)
C(9)#3-C(7)-C(8)-C(9)	1.6(9)
C(6)-C(7)-C(8)-C(9)	-177.5(5)
C(9)#3-C(7)-C(8)-C(10)	-177.7(5)
C(6)-C(7)-C(8)-C(10)	3.3(8)
C(7)-C(8)-C(9)-C(7)#3	-1.6(9)
C(10)-C(8)-C(9)-C(7)#3	177.6(5)
C(9)-C(8)-C(10)-F(4)	99.2(6)
C(7)-C(8)-C(10)-F(4)	-81.5(6)
C(9)-C(8)-C(10)-F(6)	-139.5(5)
C(7)-C(8)-C(10)-F(6)	39.7(7)
C(9)-C(8)-C(10)-F(5)	-20.9(7)
C(7)-C(8)-C(10)-F(5)	158.4(5)
Eu(01)#1-O(6)-C(11)-O(5)	-14.4(12)
Eu(01)#1-O(6)-C(11)-C(12)	164.2(5)
Eu(01)-O(5)-C(11)-O(6)	21.6(10)
Eu(01)-O(5)-C(11)-C(12)	-157.0(4)
O(6)-C(11)-C(12)-C(13)	130.5(6)
O(5)-C(11)-C(12)-C(13)	-50.7(8)
O(6)-C(11)-C(12)-C(14)#4	-52.1(7)
O(5)-C(11)-C(12)-C(14)#4	126.7(6)
C(14)#4-C(12)-C(13)-C(14)	-1.0(9)
C(11)-C(12)-C(13)-C(14)	176.4(5)
C(14)#4-C(12)-C(13)-C(15)	177.0(5)
C(11)-C(12)-C(13)-C(15)	-5.7(8)
C(12)-C(13)-C(14)-C(12)#4	1.0(9)
C(15)-C(13)-C(14)-C(12)#4	-177.1(5)
C(14)-C(13)-C(15)-F(7)	142.7(5)
C(12)-C(13)-C(15)-F(7)	-35.3(8)
C(14)-C(13)-C(15)-F(9)	22.7(8)
C(12)-C(13)-C(15)-F(9)	-155.3(5)
C(14)-C(13)-C(15)-F(8)	-96.6(6)
C(12)-C(13)-C(15)-F(8)	85.5(7)
Eu(01)-O(7)-C(16)-N(1A)	-178(4)
Eu(01)-O(7)-C(16)-N(1B)	179(3)
C(18A)-N(1A)-C(16)-O(7)	9(8)

C(17A)-N(1A)-C(16)-O(7)	-171(4)
Eu(01)-O(8)-C(19A)-N(2)	-144.6(8)
C(21A)-N(2)-C(19A)-O(8)	9.2(17)
C(20A)-N(2)-C(19A)-O(8)	177.4(11)
Eu(01)-O(8)-C(19B)-N(2)	110.7(14)
C(20B)-N(2)-C(19B)-O(8)	177.7(16)
C(21B)-N(2)-C(19B)-O(8)	0(2)
C(3)-C(4)-C(5B)-F(1A)	119.0(15)
C(2)#2-C(4)-C(5B)-F(1A)	-71(2)
C(3)-C(4)-C(5B)-F(2A)	-118.2(15)
C(2)#2-C(4)-C(5B)-F(2A)	52(2)
C(3)-C(4)-C(5B)-F(3B)	2(2)
C(2)#2-C(4)-C(5B)-F(3B)	171.8(14)
O(7)-C(16)-N(1B)-C(18B)	-6(6)
O(7)-C(16)-N(1B)-C(17B)	176(3)

Symmetry transformations used to generate equivalent atoms:
#1 -x+1,-y+1,-z+1 #2 -x+1,-y+2,-z+2 #3 -x,-y+1,-z+1
#4 -x+1,-y,-z+1

Table S6. Hydrogen bonds for Eu-TTA-*pcu* [Å and °].

D-H...A	d(D-H)	d(H...A)	d(D...A)	<(DHA)	
C(16)-H(16^a)...O(2)	0.95	2.37	2.914(8)	115.8	
C(18A^a)-H(18B^a)...O(1)#5		0.98	2.62	3.41(4)	138.4
C(18A^a)-H(18C^a)...F(4)#6		0.98	2.40	3.08(2)	126.6
C(19A^a)-H(19A^a)...O(7)0.95		2.35	2.980(11)	123.2	
C(21B^b)-H(21D^b)...O(7)0.98		2.57	3.502(19)	160.0	
C(18B^b)-H(18E^b)...O(1)#5		0.98	2.55	3.118(18)	116.9
C(18B^b)-H(18E^b)...O(8)#5		0.98	2.58	3.33(3)	134.3

Symmetry transformations used to generate equivalent atoms:
#1 -x+1,-y+1,-z+1 #2 -x+1,-y+2,-z+2 #3 -x,-y+1,-z+1
#4 -x+1,-y,-z+1 #5 -x+1,-y+1,-z+2 #6 x+1,y,z+1
